# Image of the Month: Meconium Peritonitis with Pseudocyst—A Spot Diagnosis in Newborns

**DOI:** 10.1055/s-0039-3399556

**Published:** 2020-01-28

**Authors:** Rudolph Ascherl, Duarte Vaz Pimentel, Mathias Knüpfer, Ina Sorge, Martin Lacher, Peter Zimmermann

**Affiliations:** 1 Department of Neonatology, Universitätsklinikum Leipzig, Leipzig, Germany; 2 Department of Pediatric Surgery, Universitätsklinikum Leipzig, Leipzig, Germany; 3 Department of Pediatric Radiology, Universitätsklinikum Leipzig, Leipzig, Sachsen, Germany

**Keywords:** meconium, pseudocyst, cyst, newborn, calcification

## Abstract

We report on a male preterm newborn with a large abdominal tumor found on prenatal ultrasound 2 weeks prior to delivery at 36 + 0 weeks of gestation. A postnatal abdominal plain film showed a mass with well-defined rim calcifications (“eggshell”), suggestive of a meconium pseudocyst. On the 4th day of life, the boy underwent exploratory laparotomy with resection of the cyst and end-to-back jejunojejunostomy. The postoperative course was uneventful. A meconium pseudocyst is the correlate of a sterile peritonitis caused by antenatal bowel perforation. It is an easily recognizable spot diagnosis any pediatrician and pediatric surgeon should be aware of.

## Introduction

Meconium peritonitis is a rare type of sterile peritonitis caused by antenatal bowel perforation with spillage of meconium into the peritoneal cavity. 1 The estimated prevalence is 1 per 30,000 live births, and mortality ranges from 11 to 50%. 2 The bowel perforation may have different causes including atresia, stricture, meconium ileus, internal hernia, volvulus, intussusception, duplication, or Meckel's diverticulum. 2 3 Meconium peritonitis leads to inflammation and fibrosis (fibroadhesive-type) and can be complicated by the presence of pseudocysts (cystic-type). 4 The typically rim-calcified cysts lack an intestinal epithelial layer due to inflammation by digestive enzymes. Usually a small muscle sheath connects the cyst to the rest of the bowel. 2

## Case Report

A 34-year-old woman, gravida 2, para 1, was referred from an outside hospital with premature rupture of membranes and contractions. Two weeks before an abdominal mass of unknown origin was detected on routine ultrasound. After 36 + 0 weeks of gestation, a male preterm newborn of 3,410 g (P _89_ ) was spontaneously delivered.

**Fig. 1:**
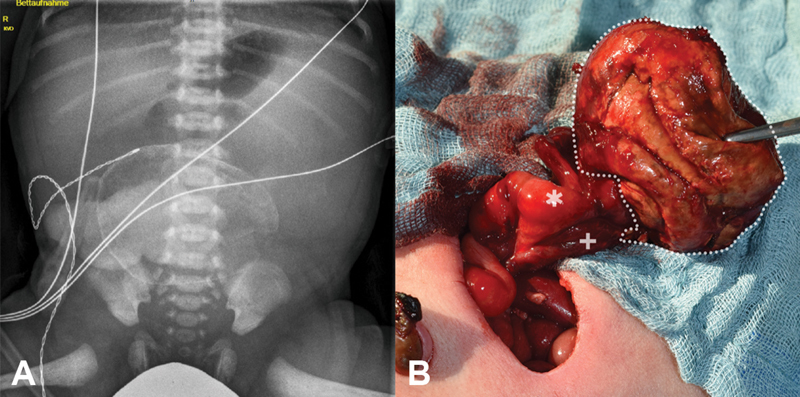
Preoperative plain abdominal film (A) and intraoperative findings (B). Meconium pseudocyst, the dilated prestenotic jejunum (*), and unused small poststenotic jejunum (+) at the base of the pseudocyst. Note the substantial caliber difference.

**Fig. 2:**
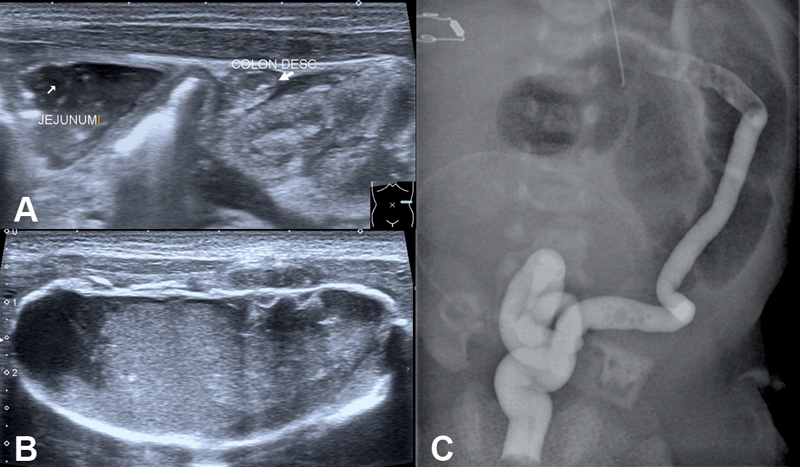
Ultrasound and contrast enema. ( **A** ) Ultrasound of the meconium pseudocyst with an echogenic rim and heterogenous content. ( **B** ) In the left abdomen were grossly distended intestinal loops, the descending colon appears empty. ( **C** ) Contrast enema shows an unused microcolon.

Despite the palpable abdominal mass, the boy seemed not be affected. At 2 hours of life, an abdominal plain X-ray showed dilated intestinal loops in the upper abdomen and an “eggshell” oval calcification suggestive of a meconium pseudocyst ( Fig. 1A ). On ultrasound ( Fig. 2A and B ), the mass (6 × 5 × 3 cm) had an echogenic wall without increased perfusion and a heterogeneous content. In the left upper abdomen were grossly distended intestinal loops with a to-and-fro peristalsis, the colon appears empty. On contrast enema the colon appeared small and unused without any stricture or atresia ( Fig. 2C ). A nasogastric tube was placed, and the patient was scheduled for surgery.

On the 4th day of life, the patient underwent explorative laparotomy: A large meconium pseudocyst and a small part of obstructed bowel were removed ( Fig. 1B ). With a size discrepancy of 7:1, a single layer end-to-back anastomosis between the two ends of the jejunum was performed. In addition, a large percutaneous central venous catheter was placed to facilitate parenteral nutrition. Further laboratory workup did not confirm cystic fibrosis as a potential cause of meconium peritonitis. Enteral feeds were started on the fifth postoperative day and advanced slowly until full feeds on the 28th day of life. The patient was discharged on breastfeeding with a weight of 3,750 g. On follow-up after 3 months, the child was thriving.

## Discussion

Meconium pseudocyst is the result of meconium peritonitis where adjacent intestinal loops adhere to the meconium leak. 2 Like in our patient, the bowel distal to the pseudocyst is usually unused and of small caliber. 3 4

There are many differential diagnoses for intraabdominal calcifications in the newborn period, most of them neoplastic. 5 However, “eggshell calcifications” are pathognomonic for meconium pseudocysts. These calcifications are thought to be caused by a chemical reaction of pancreatic enzymes within the extravasated meconium. Their presence speaks against (but does not rule out) cystic fibrosis, which is present in 15 to 40% of children with meconium peritonitis in Caucasians. 6 Resection of the pseudocyst as well as the segment of small bowel followed by an enteral anastomosis is the treatment of choice. 3 4

## Conclusion

Meconium pseudocysts are easy to recognize and an important radiologic spot diagnosis that every pediatrician and pediatric surgeon should be aware of. Resection of the pseudocyst is the therapy of choice and can be undertaken electively in the first days of life.

## Notes

New Insights & the Importance for the Pediatric Surgeon Meconium pseudocyst is a rare entity that can be immediately diagnosed with an abdominal X-ray. Every doctor dealing with newborns should be able to recognize it.
